# Two New 1D Supramolecular Compounds Based on PbI_2_ for Efficient Iodine Capture

**DOI:** 10.3390/molecules28072934

**Published:** 2023-03-24

**Authors:** Xingxing Zhang, Jian Li, Yunyin Niu

**Affiliations:** 1 Green Catalysis Center, College of Chemistry, Zhengzhou University, Zhengzhou 450001, China; 2 College of Ecology and Environment, Zhengzhou University, Zhengzhou 450001, China

**Keywords:** supramolecular compounds, adsorbent, iodine capture

## Abstract

Two new inorganic-organic hybrid crystals based on PbI_2_ were assembled through the solvent evaporation method, namely, {[L1]·[Pb_2_I_6_]}_n_ (**1**) and {[L2]_2_·[Pb_3_I_10_]}_n_ (**2**). L1-L2 are a series of multivalent nitrogen-containing cationic ligands. Compounds **1**–**2** were characterized by single-crystal X-ray diffraction, elemental analysis, FT-IR, powder X-ray diffraction, and thermogravimetric microanalysis. The results showed that the adsorption rate of 80 mg compound **1** to iodine reached 96.59%, indicating a high iodine capture performance in cyclohexane solution. In the meantime, the adsorption kinetics is most suitable for a pseudo-second-order model, and the adsorption process is mainly chemisorption. Adsorption thermodynamics is most suitable for the Langmuir model, indicating that adsorption occurs on the surface of the monolayer. According to the adsorption mechanism, it can be inferred that the structure of compound **1** contains amino, benzene, N heterocyclic, and other active groups, that is, indirectly increases the adsorption site with iodine, and the chemical reaction with iodine improves the removal rate of iodine in cyclohexane solution. In addition, compound **1** was found to have good iodine adsorption and recyclability by cyclic experiments. Therefore, the synthesized compound **1** can be used as a potential and excellent iodide capture adsorbent, which may have a good application prospects in the future.

## 1. Introduction

In the foreseeable future, nuclear energy as a green, clean, and sustainable emerging energy source will continue to provide energy and attract more widespread attention. However, the tragic cost of the Fukushima nuclear accident in Japan in 2011 once again aroused worldwide attention toward the disposal of nuclear waste [1], especially the disposal of radioactive iodine produced in the process of nuclear fission [2]. With an extremely long half-life of 1.57 × 10^7^ years, ^129^I poses a long-term environmental risk due to bioaccumulation [3,4]. In contrast, ^131^I has a shorter half-life of 8.02 days, but it poses a significant risk to human health [5,6,7,8]. Because both ^129^I and ^131^I are highly volatile, they can quickly disperse in the air and water and destroy the earth’s ecosystem. Through the enrichment of the food chain, radioactive iodine continues to enter the human body and participate in human metabolism, causing thyroid damage and even cancer [9,10,11]. In addition, radioactive iodine combined with volatile organic materials can cause irreversible damage to the central nervous system [12]. Moreover, many chemical and pharmaceutical enterprises are also facing the problem of iodine wastewater and waste gas treatment. Therefore, it is of great theoretical and practical significance to explore and develop safe, efficient, and economical repositories to capture and isolate ions and neutral iodine residues [13].

Adsorption technology is a simple way to reconcentrate pollutants, and it remains one of the most hopeful means to reduce the release of iodine [14]. At present, the most common method of iodine capture that has been developed and adopted is the solid capture method. The applied solid adsorbents are mainly activated carbon, silica, silver-based materials, metal-organic framework, etc. [15,16,17]. However, these adsorbents are limited by some factors. Although activated carbon and silica have the advantages of high capacity and availability [10], their limited accessible surface area leads to a low capacity of iodine capture. Recently, researchers have discovered that silver-based materials [18] can be used for vapor iodine capture and storage, but silver cannot be widely used due to its high price, strong toxicity, and limited adsorption capacity [19]. Metal-organic skeleton materials are unstable under wet conditions. Therefore, the development of novel adsorbents with higher thermal and chemical stability for iodine capture remains a challenge.

In recent years, supramolecular chemistry has developed rapidly. A large number of inorganic–organic hybrid crystals with novel structures have been reported, and researchers began to pay attention to the improvement of their practical application value [20,21,22,23,24,25,26,27]. Supramolecular materials combine the stability of inorganic materials and the adjustability of organic materials, which gives organic–inorganic hybrid materials unique advantages in the field of iodine molecular enrichment [12]. Some electron-rich groups, such as −NH, −NH_2_, −C=N−, heterocyclic (S, P), aromatic ring, and so on, can adsorb electron-deficient I_2_ by forming charge transfer complexes, so as to improve the adsorption capacity and adsorption rate by adjusting the adsorption dynamics between organic–inorganic hybrid materials modified with electron-rich functional groups and iodine [28]. Our research group has been engaged in the preparation and performance research of supramolecular compounds for a long time [29,30,31,32,33,34,35,36,37]. Herein, we attempt to get compounds with certain expected structures through a self-assembly reaction between self-designed organic ligands and selected metal sources and use them to study the adsorption of iodine.

**Scheme 1:**
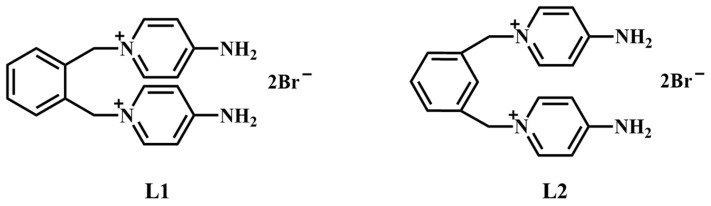
Structures of L1 and L2.

The choice of ligands has a crucial influence on the structure and function of compounds. In the design of organic ligands, we consider multivalent nitrogen-containing cationic ligands L1–L2 (Scheme 1). The introduction of amino functional groups into the ligand is beneficial to enhance the affinity between the adsorbent and iodine, thus improving its iodine adsorption capacity. In recent years, lead iodide (PbI_2_) has attracted much attention because of its high atomic coefficient, wide band gap, high resistivity, and carrier mobility lifetime, which make it an ideal material for the preparation of room-temperature X-ray and γ-ray detectors. Lead iodide crystal is a promising material for room-temperature nuclear radiation detectors. As a new type of room-temperature nuclear radiation detector, it can be widely used in the fields of environmental monitoring, nuclear medicine, nondestructive testing, nuclear weapons penetration, aerospace, astrophysics, and high-energy physics [38,39,40]. In this research, we used PbI_2_ as the selected metal source to react with multivalent nitrogen-containing cationic ligands (L1–L2) to obtain compounds **1**–**2** by solvent evaporation at room temperature. We used compounds **1**–**2** as adsorbents to explore the iodine adsorption performance in cyclohexane solution. It is worth mentioning that compound **1** exhibits high iodine adsorption performance.

## 2. Results and Discussion

### 2.1. Crystals Structures of Compounds **1**–**2**

**Figure 1:**
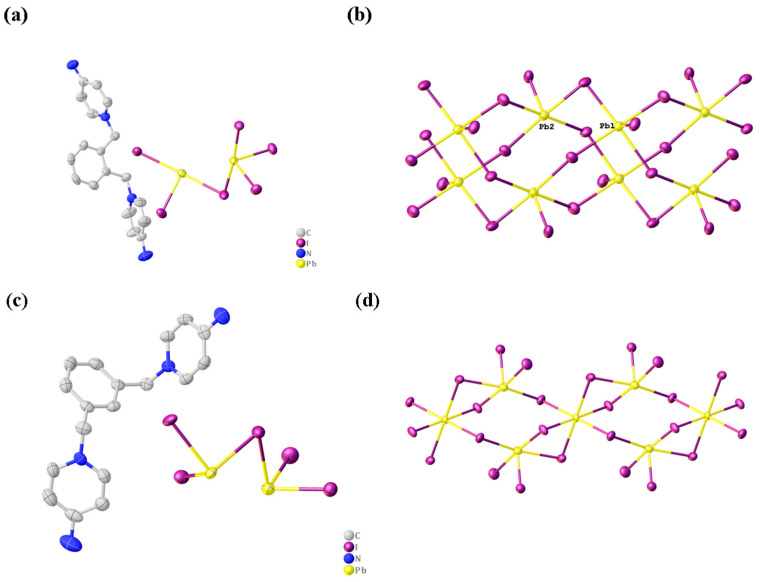
(**a**) The smallest asymmetric unit of compound **1**; (**b**) The anion cluster of compound **1**; (**c**) The smallest asymmetric unit of compound **2**; (**d**) The anion cluster (**b**) of compound **2** (with the H atom omitted).

The results of X-ray single crystal diffraction for compounds **1**–**2** are shown in Figure 1. Compound **1** belongs to the *P*2_1_*/n* space group in a monoclinic crystal system. The structural unit diagram of compound **1** is shown in Figure 1a, consisting of one crystallographically independent [L1]^2+^, and cluster anion [Pb_2_I_6_]^2−^. The Pb-I bond lengths range from 2.9931(5) Å to 3.3275(5) Å. Compound **2** belongs to the *P*2_1_*/c* space group in a monoclinic crystal system. The structural unit diagram of compound **2** is shown in Figure 1c, consisting of one crystallographically independent [L2]^2+^, and cluster anion [Pb_1.5_I_5_]^2−^. The inorganic portion of compounds **1**–**2** is a different 1D anion chain formed by anion clusters connected to each other by I-Pb bonds, as shown in Figure 1b,d. Although compounds **1**–**2** were obtained from similar ligands under the same conditions, Pb^2+^ in compounds **1**–**2** showed different coordination modes. In compound **1**, Pb^2+^ has only one coordination mode of five coordination, while in compound **2**, Pb^2+^ has two coordination modes of five and six coordination. So the cationic ligands L1 and L2 exhibit different template effects.

### 2.2. Structure Characterizations of Compounds **1**–**2**

**Figure 2:**
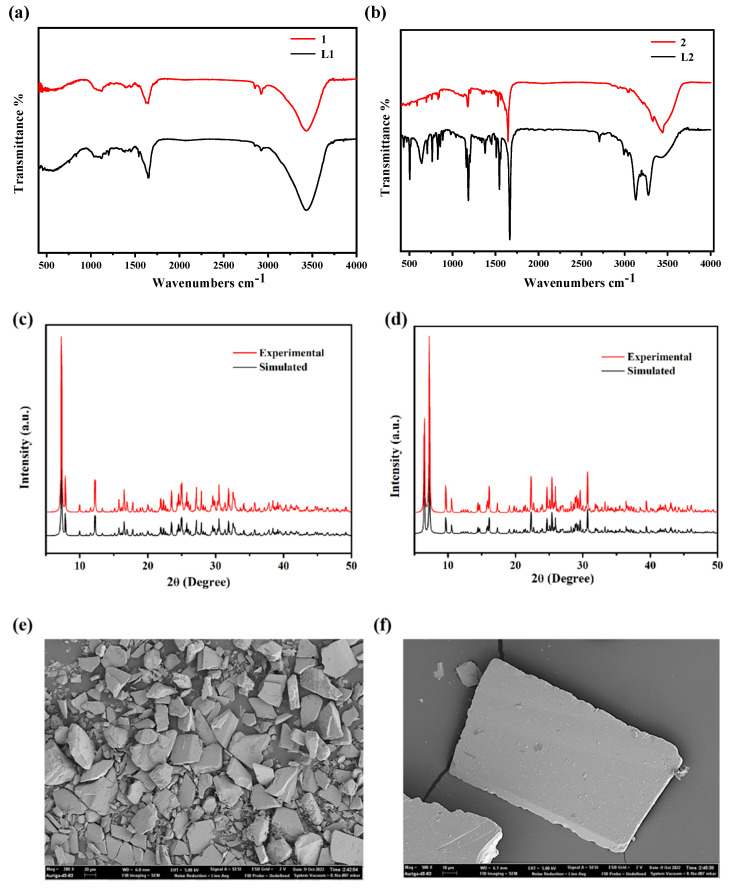
(**a**) The FT−IR spectrum of compounds **1**; (**b**) The FT−IR spectrum of compounds **2**; (**c**) PXRD pattern of compounds **1**; (**d**) PXRD pattern of compounds **2**; (**e**) The SEM crystal diagram of compound **1** at 200× magnification; (**f**) The SEM crystal diagram of compound **1** at 500× magnification.

Fourier-transform infrared (FT-IR) spectra were used to study the functional groups of compounds **1**–**2**. The FT-IR spectrum of the synthesized ligands L1–L2 and compounds **1**–**2** is distinctly revealed in Figure 2a,b. The characteristic peaks of compounds **1** and **2** are consistent with the corresponding ligands (L1–L2). The broad peak at nearly 3420 cm^−1^ corresponded to the N-H stretching mode, and the signal peaks appearing around 1675 cm^−1^, 1587 cm^−1^, and 1456 cm^−1^ were attributed to the stretching vibration of the aromatic ring. In addition, the signal peaks present around 2950 cm^−1^ are vibrational stretching peaks with saturated C-H. Powder X-ray diffraction (PXRD) patterns of compounds **1**–**2** were recorded and compared with the simulated single-crystal diffraction data in order to affirm the purity of the compounds. As shown in Figure 2c,d, the positions of the peaks are basically consistent with the simulated patterns generated from the results of the single crystal diffraction data, indicating the purity of products. Scanning electron microscopy (SEM) is used to study the morphology of compound **1**, as shown in Figure 2e,f, compound **1** presents a regular block structure.

**Figure 3:**
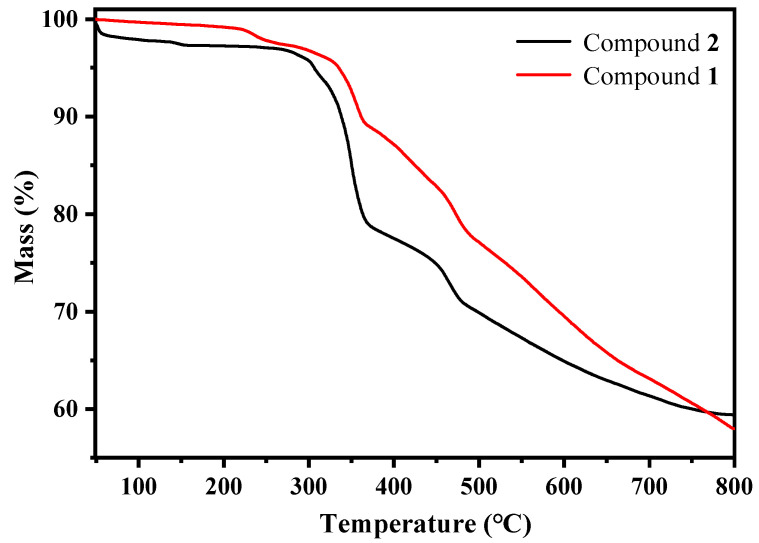
The TGA curve of compounds **1** and **2**.

A thermogravimetric analysis (TGA) was conducted under air atmosphere conditions to test the thermal stability of compounds **1**–**2**. The results of the TGA analysis are shown in Figure 3, and the thermogravimetric decomposition processes of compounds **1**–**2** were different. The thermogravimetric decomposition of compound **1** is divided into three stages. Compound **1** remains basically unchanged from room temperature to 250 °C. The mass loss between 250 and 320 °C can be attributed to the decomposition of organic cations; the mass loss between 320 and 800 °C may be caused by the collapse of the inorganic anion skeleton. In addition, the thermogravimetric decomposition of compound **2** is divided into four stages. Compound **2** remains basically unchanged from room temperature to 300 °C. The mass loss between 300 and 360 °C can be attributed to the decomposition of organic cations; and due to the fact that Pb^2+^ in compound **2** has two coordination modes of five and six coordination, the mass loss between 360 and 470 °C and 470 and 800 °C may be caused by the collapse at the different inorganic skeletons with different Pb^2+^ coordination environments. It can be found that compound **2** is more thermally stable than compound **1**.

### 2.3. Capture of Iodine in Cyclohexane Solution

**Figure 4:**
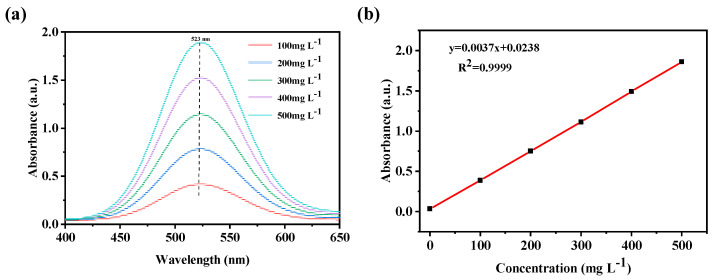
(**a**) UV−vis spectra of cyclohexane solutions with different iodine concentrations; (**b**) Standard curve of cyclohexane solution of iodine at 523 nm.

In order to avoid a polar solvent interfering with the host–guest interaction between the compounds and iodine, we chose to study the adsorption effect of compounds on iodine in a nonpolar cyclohexane solvent solution. First, UV-vis was used to determine the standard curve of the cyclohexane solution of iodine, and the results were shown in Figure 4. It can be seen that the maximum absorption wavelength of the cyclohexane solution of iodine is 523 nm. Therefore, we use the absorbance of the iodine solution at 523 nm as the standard curve for the concentration. The standard curve is y = 0.0037x + 0.0238, and the correlation coefficient R^2^ is 0.9999.

**Figure 5:**
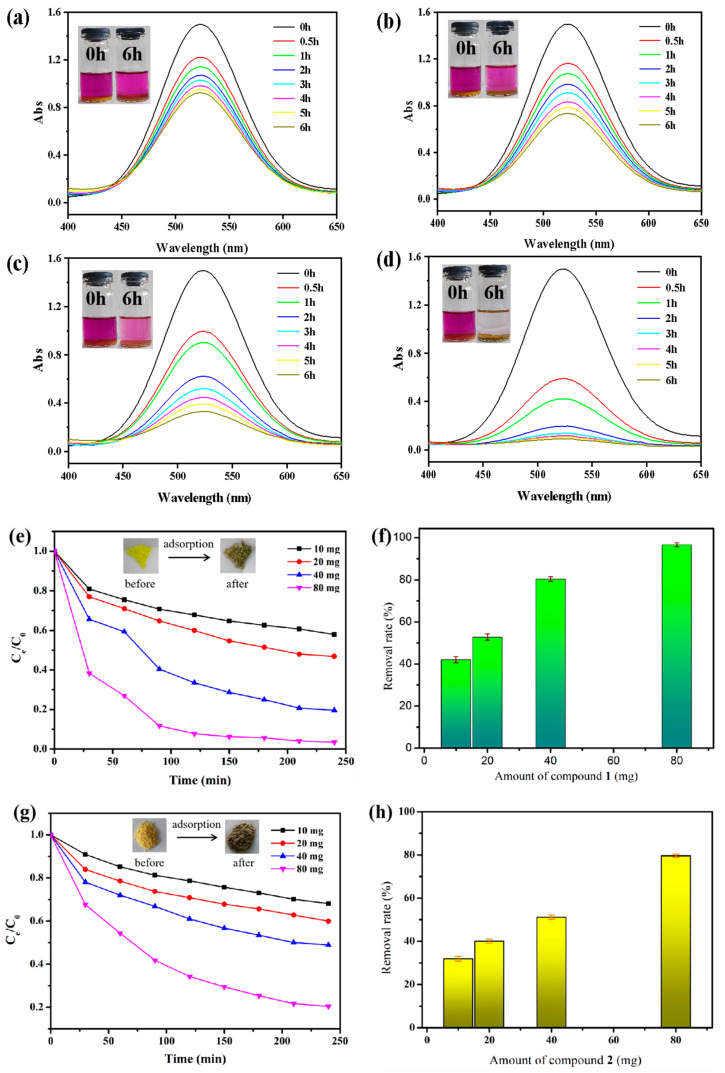
(**a**) Uv-vis spectrogram and color change of iodine solution when 10 mg compound **1** is added; (**b**) Uv-vis spectrogram and color change of iodine solution when 20 mg compound **1** is added; (**c**) Uv-vis spectrogram of and color change iodine solution when 40 mg compound **1** is added; (**d**) Uv-vis spectrogram and color change of iodine solution when 80 mg compound **1** is added; (**e**) Effect of different compound **1** dosages on iodine degradation; (**f**) Removal rate of iodine degraded by compound **1**; (**g**) Effect of different compound **2** dosages on iodine degradation; (**h**) Removal rate of iodine degraded by compound **2**.

In order to analyze the iodine adsorption effect of the synthesized compounds, the influence of the amount of adsorbent on the iodine removal efficiency was studied. Compounds at different doses (10 mg, 20 mg, 40 mg, 80 mg) were added to the iodocyclohexane solution. After being placed at room temperature for 6 h, it was found through the experimental phenomenon that the iodocyclohexane solution was observed to be colorless from imperial purple as the adsorption time went on (Figure 5a–d). Compounds **1**–**2** changed from yellow to dark green after absorbing iodine. The concentration of iodine solution was determined by UV-VIS spectroscopy (Figure 5e,g), and the removal rates of iodine were calculated. The corresponding iodine removal rates by compound **1** were 42.07%, 53.14%, 80.40%, and 96.59% (Figure 5f); and the adsorption removal rates of compound **2** for iodine were 31.89%, 40.08%, 51.14%, and 79.6%. The comparative experimental results showed that compound **1** had a better adsorption effect on iodine than compound **2**. From the structure of the compounds, it was inferred that Pb^2+^ in compound **1** had only one five-coordinated mode, while Pb^2+^ in compound **2** had two coordination modes: five and six-coordination. In compound **2**, Pb^2+^ has more coordination modes for the iodine atom, which leads to that compound **2** having a less effective adsorption effect on iodine molecules than compound **1**. Therefore, relatively speaking, compound **1** has better adsorption performance for iodine, and 80 mg compound **1** has the best adsorption effect on iodine, which may be because the number of active sites in contact with iodine increases with the increase in the number of adsorbents, to improve the adsorption efficiency. At the same time, after adding 80 mg compound to absorb iodine, the concentration of residual iodine in the solution reached 13.64 mg/L. It can be found that the concentration of residual iodine after the treatment of compound **1** is very low. The adsorption efficiency of compounds **1**–**2** is comparable with some reported two-dimensional materials in cyclohexane solution. Zhang et al. successfully prepared a unique thorium MOF (metal-organic framework) ThTTHA with a thorium oxide wheel cluster by using thorium nitrate and a semirigid triazine hexarboxylic acid ligand H_6_TTHA (H_6_TTHA = 1, 3, 5-triazine-2, 4, 6-triamine hexaacetic acid) under solvothermal synthesis. After 6 h adsorption in cyclohexane, the removal efficiency of iodine by 100 mg Th-TTHA reached 50.75% [41]. Zhang et al. also synthesized a novel uranyl-organic framework [(CH_3_)_2_NH_2_][UO_2_(TATAB)]·2DMF·4H_2_O (U-TATAB), and it was assembled through the solvothermal synthesis of UO_2_(CH_3_COO)_2_·2H_2_O and triazine tricarboxylate linker H_3_TATAB (H_3_TATAB = 4,4′,4″-s-triazine-1,3,5-triyltri-p-aminobenzoate), After 72 h of adsorption in cyclohexane, the maximum removal efficiency of iodine by U-TATAB reached 98.8% [42]. Li et al. synthesized two novel thorium-based metal−organic frameworks (MOFs), Th-SINAP-7 and Th-SINAP-8, by solvent-thermal reaction of thorium nitrate with 1,4- or 2,6-naphthalenedicar-boxylic acid in the presence of acid modulators. After 24 h adsorption, it was found that Th-SINAP-8 could remove more than 99% of iodine, and Th-SINAP-7 could remove 86.84% of iodine [43].

**Figure 6:**
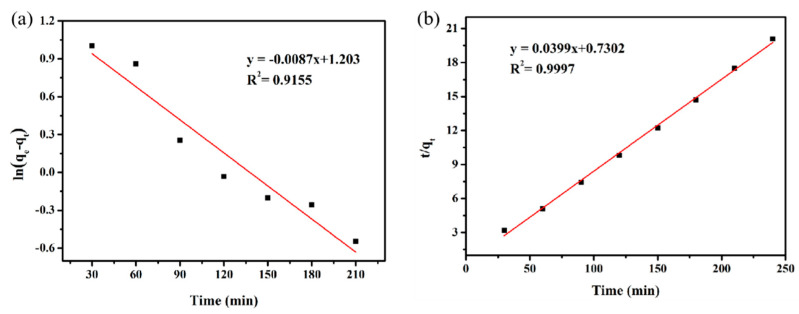
(**a**) The illustration of pseudo−one−order kinetics for the adsorption process of iodine by compound **1** in cyclohexane solution; (**b**) The illustration of pseudo−second−order kinetics for the adsorption process of iodine by compound **1** in cyclohexane solution.

Through experimental study, it was found that compound **1** had a better adsorption effect on iodine than compound **2**. In order to further investigate the adsorption mechanism and performance of iodine by compound**s**, the iodine adsorption data of 80 mg of compound **1** were selected at different times, and the quasi-first order kinetic model and quasi-second order kinetic model were used to fit. The fitting data are shown in Figure 6a,b, and the correlation coefficient is shown in Table 1. The comparison of R^2^ showed that the adsorption kinetics of compound **1** was more in line with the pseudo-second-order kinetic model, indicating that the adsorption of iodine by compound **1** was controlled by the chemisorption process, which may be due to the charge transfer between electron-rich group -NH_2_ in the structure of compound **1** and electron-lacking I_2_, that is, the adsorption power is generated to improve the adsorption capacity and adsorption rate.

**Figure 7:**
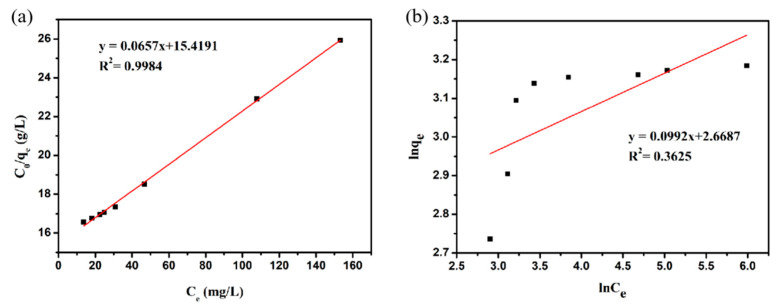
(**a**) The adsorption isotherms of Compound **1** for iodine were fitted to Langmuir Freundlich; (**b**) The adsorption isotherms of Compound **1** for iodine were fitted to Freundlich models.

Langmuir and Freundlich curve models were used to fit the iodine adsorption data of compound **1**. The obtained linear fitting curve is shown in Figure 7a,b, and the adsorption constants and correlation coefficients are listed in Table 2. According to the value of R^2^, it can be concluded that the Langmuir model can better describe the adsorption of iodine by compound **1** than the Freundlich model, indicating that the adsorption of iodine by compound **1** is mainly on the surface of the monolayer.

### 2.4. Cyclic Experiment

**Figure 8:**
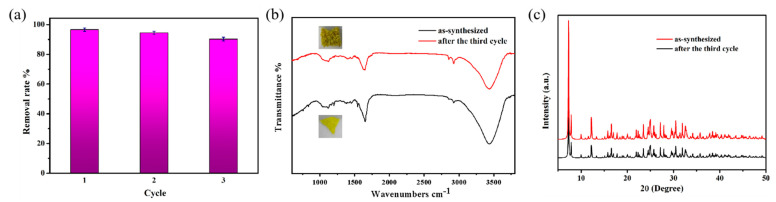
(**a**) Cycle test of adsorption of iodine by compound **1**; (**b**) FT−IR spectra and colors change of compound **1** before and after iodine adsorption; and (**c**) PXRD patterns of compound **1** before and after iodine adsorption.

The recyclability of materials is an important criterion to evaluate whether materials have application value. After the removal of iodine by adsorption with compound **1** each time, the adsorbent was collected, and then anhydrous ethanol was used as the clean solution to remove the adsorbent. The collected adsorbent was put into fresh iodo-cycloethane solution to start a new cycle. In the cycle experiment, all experimental conditions are exactly the same as in the first cycle experiment. After three times of adsorption-desorption experiments, the results are shown in Figure 8a; the removal efficiency can still reach 90.27% after the third cycle (96.59% in the first round). For a small decrease in removal rate, it can be assumed that a small number of active sites are incompletely resolved. The removal rate of iodine from compound **1** remained high after each cycle. In addition, the adsorbent was characterized by FT-IR and PXRD after three cycles. As shown in Figure 8b,c, compared with fresh samples, FT-IR peaks before and after the adsorption reaction showed almost no difference. The intensity of the characteristic peaks of PXRD decreased to different degrees, but the characteristic peaks were still observed, which was speculated to be due to the adhesion of iodine on the surface of the adsorbent after adsorption. In conclusion, compound **1** has good recyclability and has objective application prospects.

## 3. Materials and Methods

### 3.1. Materials

O-dibenzyl bromide, M-dibenzyl bromide, 4-Aminopyridine, PbI_2_, KI, methanol, acetonitrile, and I_2_ (Sinopharm Chemical Reagent Co., Ltd, 52 Ning Bo Road, Shanghai 200002, China) were all used in the experiment. All the reagents of AR grade were used without any further purification. The ligands (L1–L2) were prepared on the basis of the methods in the literature [44,45,46].

### 3.2. Synthesis Methods

#### 3.2.1. Synthesis of {[L1]·[Pb_2_I_6_]}_n_ (**1**)

In the presence of excess KI, PbI_2_ (0.0046 g, 0.01 mmol) was dissolved in MeCN/H_2_O. With stirring, a solution of L1·Br_2_ (0.0045 g, 0.01 mmol) in CH_3_OH/H_2_O was added to the above solution. The mixture was stirred for 10 min. The resulting mixture was filtered and left standing at room temperature for 2 days to give yellow block crystals, yield 57%. Anal. Calcd for C_18_H_20_I_6_N_4_Pb_2_ (1468.16): C, 14.71; H, 1.36; N, 3.81. Found: C, 14.75; H, 1.38; N, 3.83. IR (KBr, cm^−1^): 3746.01 (w), 3435.02 (m), 2923.59 (w), 2852.76 (w), 1646.65 (m), 1453.25 (w), 1398.75 (w), 1121.80 (w), 519.48 (w), 466.89 (w), 441.53 (w).

#### 3.2.2. Synthesis of {[L2]_2_[Pb_3_I_10_]}_n_ (**2**)

In the presence of excess KI, PbI_2_ (0.0046 g.0.01 mmol) was dissolved in MeCN/H_2_O. With stirring, a solution of L2·Br_2_ (0.0045 g, 0.01 mmol) in MeCN/H_2_O was added to the above solution. The mixture was stirred for 10 min. The resulting mixture was filtered and left standing at room temperature for 1 week to give yellow block crystals, yield 30%. Anal. Calcd for C_36_H_40_I_10_N_8_Pb_3_ (2475.33): C, 17.45; H, 1.62; N, 4.52. Found: C, 17.41; H, 1.65; N, 4.55. IR (KBr, cm^−1^): 3441.37 (m), 3329.22 (m), 3036.76 (w), 1645.96 (m), 1560.75 (m), 1528.59 (m), 1506.60 (w), 1451.40 (m), 1368.97 (w), 1348.20 (m), 1207.92 (w), 1177.10 (m), 1122.50 (m), 844.32 (m), 834.64 (m), 760.96 (m), 697.56 (m), 589.35 (m).

### 3.3. Characterization Methods

Fourier-transform infrared spectrum (FT-IR) was measured on a Nexus870 spectrophotometer (Nicolet, Thermo Fisher Scientific, Walthamm, MA, USA), in the form of a KBr disk (4000–400 cm^−1^). Elemental analyses (C, H, and N) were performed on a FLASH EA 1112 elemental analyzer. The morphologies of the compound were examined using scanning electron microscopy (SEM) on a JSM-6510LV unit (Jeol, Tokyo, Japan). A Netzsch equipment model STA 449 F3 Jupiter (Germany) thermal analyzer was used to record simultaneous TG curves in flowing air atmosphere of 20 mL·min^−1^ at a heating rate of 5 °C·min^−1^ in the temperature range 25–800 °C using platinum crucibles. Powder X-ray diffraction (PXRD) data were measured on a Riguku DMAX2550 diffractometer (Tokyo, Japan) (Cu-Kα, λ = 1.5418 Å) with an X’ Celerator detector. Simulation of the PXRD spectra was carried out by the single-crystal data and diffraction-crystal module of the Mercury (Hg) program (Version 001.000) available free of charge via the Internet at http://www.iucr.org (accessed on 15 February 2023) at any time. Suitable single crystals of **1**–**2** were carefully selected under an optical microscope and glued to thin glass fibers. The crystallographic data of compounds are on a Bruker APEX-II area detector diffractometer equipped with graphite monochromatic Cu-Kα radiation (λ = 1.54184 Å) at 293(2) K acquired. The structure was refined with full-matrix least-squares techniques on F^2^ using the OLEX2 program package. The CCDC reference numbers are 2,207,966 and 2,207,967 for compounds **1** and **2,** respectively. The crystallographic data are listed in Table 3. The selected bond distances (Å) and angles (deg) are presented in Table 4.

### 3.4. Adsorption Experiment

#### 3.4.1. Iodine Sorption Study

In order to study the iodine adsorption capacity of the compounds in cyclohexane solution, a simulation experiment was conducted. The specific experimental process is as follows: Firstly, 500 mg·L^−1^ iodo-cyclohexane solution was prepared, 80 mg compound was added into 10 mL iodo-cyclohexane solution, and the solution was standing at room temperature. The color change of iodo-cyclohexane solution was observed during adsorption, and the removal rate of iodine was evaluated by UV-VIS spectrum. The iodine removal efficiency (*RE*; %) and the adsorption capacity (*q_e_*; mg·g^−1^) of the compound were calculated by Equations (1) and (2), respectively [47].

(1) $$RE(\%)=\left(1-\frac{C_{t}}{C_{0}}\right)\times 100\%$$

(2) $$q_{e}=\frac{\left(C_{0}-C_{e}\right)\times V}{m}$$

where *C_0_* is the initial concentrations (mg·L^−1^) of iodine; *C_e_* is the equilibrium concentrations (mg·L^−1^) of iodine; *C_t_* is the iodine concentrations (mg·L^−1^) at time *t*; *V* is the volume (L) of iodo-cyclohexane solution; and *m* is the compound weight (g).

#### 3.4.2. Kinetic Studies

In order to further understand the adsorption of iodine by compounds, the pseudo-first-order kinetic model and pseudo-second-order kinetic model were used to fit the experimental results and determine the actual situation of iodine adsorption. The nonlinear forms of the quasi-first-order and quasi-second-order dynamics models are defined by Equations (3) and (4) [48], respectively:

(3) $$\mathrm{lg}(q_{e}-q_{t})=-\frac{K_{f}}{2.303}t+\mathrm{lg}q_{e}$$

(4) $$\frac{t}{q_{t}}=\frac{1}{q_{e}}t+\frac{1}{K_{s}q_{e}^{2}}$$

where *q_e_* is the equilibrium adsorption capacity (mg·g^−1^) of the adsorbent; *q_t_* is the adsorption capacity (mg·g^−1^) of the adsorbent at time *t*; *K_f_* is the pseudo-first-order model rate constants of adsorption (min^−1^); *K_s_* is the pseudo-second-order model rate constants of adsorption (g·mg^−1^·min).

#### 3.4.3. Models of Adsorption Isotherm

The adsorption isotherm is very important to understand the nature of the specific relationship between adsorbing material and adsorbent. Langmuir’s isothermal adsorption model is a single molecular layer adsorption model, and Freundlich’s isothermal adsorption model is a multi-molecular layer adsorption model. In this experiment, two models were fitted according to the iodine adsorption experiment data of compound **1**: Langmuir (5) and Freundlich (6) isotherm adsorption model [49].

(5) $$\frac{C_{0}}{q_{e}}=\frac{1}{q_{m}K_{1}}+\frac{C_{e}}{q_{m}}$$

(6) $$\ln q_{e}=\ln K_{2}+\frac{1}{n}\cdot \ln C_{e}$$

where *q_m_* is the maximum adsorption capacity (mg·g^−1^) of the adsorbent; *K*_1_ is the Langmuir adsorption constant (L·mg^−1^); *K*_2_ is the Freundlich adsorption constant (g·mg^−1^·min^−1^); *n* is a constant that characterizes the surface heterogeneity, and constant is always greater than 1.

## 4. Conclusions

In summary, two new supramolecular compounds **1**–**2** were synthesized through the solvent evaporation method. They were characterized by single-crystal X-ray diffraction, elemental analysis, Fourier-transform infrared spectrum, powder X-ray diffraction, and thermogravimetric analysis. Experimental results show that compound **1** exhibits high iodine capture performance in cyclohexane solution. The removal rate of iodide from cyclohexane solution reached 96.59% with 80 mg of compound **1**. We speculated that the synthesized compound **1** contained amino, benzene, N heterocyclic, and other active groups, which could interact with iodine and improve the ability of compound **1** to capture iodine in cyclohexane solution, indicating that the synthesized material has a good application prospect for iodine adsorption. The mechanism of iodine adsorption by compound **1** was investigated, and it is found that the adsorption kinetics fit the quasi-first-order model best, and adsorption thermodynamics fit the Langmuir model best. The synthesized compound **1** can be used as a potential and excellent iodine capture adsorbent. In the subsequent study, we can explore the adsorption performance of compound **1** on other pollutants. The capture experiments of iodine in solution with various ions and materials similar to the real sample and in an aqueous solution are on the way.

## Figures and Tables

**Table 1:** Parameter values of pseudo−first−order and pseudo−second−order adsorption kinetics equation.

Sample	Pseudo−First−Order−Model			Pseudo−Second−Order−Model		
	*qe* (cal) (mg·g^−1^)	*Kf* (1·min−1)	R^2^	*qe* (cal) (mg·g^−1^)	*Ks* (g·mg−1·min−1)	R^2^
Compound **1**	15.96	0.0176	0.9155	24.96	0.0022	0.9997

**Table 2:** The parameters calculated from Langmuir and Freundlich models.

Sample	Langmuir			Freundlich		
	*q_m_* (mg·g^−1^)	*K*_1_ (L·mg^−1^)	R^2^	1/n	*K*_2_ (g·mg^−1^·min^−1^)	R^2^
Compound **1**	15.22	0.0043	0.9984	0.0992	466.33	0.3625

**Table 3:** Crystal structure data of Compounds **1**–**2**.

Compounds	1	2
Formula	C_18_H_20_I_6_N_4_Pb_2_	C_36_H_40_I_10_N_8_Pb_3_
Formula Weight	1468.16	2475.33
Temperature/K	299.00	300.00
Crystal system	Monoclinic	Monoclinic
Space group	P2_1_/n	P2_1_/c
a/Å	9.4089 (2)	13.724 (3)
b/Å	22.4783 (5)	24.503 (5)
c/Å	14.4369 (4)	8.9479 (18)
α/°	90	90
β/°	93.4570(10)	94.487 (7)
γ/°	90	90
Volume/Å^3^	3047.79 (13)	2999.8 (10)
Z	4	2
ρ calc, g/cm^3^	3.200	2.740
μ/mm^−1^	17.124	13.574
F(000)	2552.0	2176.0
Crystal size/mm^3^	0.26 × 0.23 × 0.14	0.23 × 0.22 × 0.21
Reflections collected	76,647	78,864
Independent reflections	7001[R_int_ = 0.0831, R_sigma_ = 0.0397]	6848[R_int_ = 0.0841, R_sigma_ = 0.0359]
Data/restraints/parameters	7001/0/272	6848/0/261
Goodness-of-fit on F^2^	1.027	1.077
Final R indexes (I ≥ 2σ (I))	R_1_ = 0.0302, wR_2_ = 0.0629	R_1_ = 0.0301, wR_2_ = 0.0638
Final R indexes (all data)	R_1_ = 0.0423, wR_2_ = 0.0662	R_1_ = 0.0454, wR_2_ = 0.0672
Largest diff. peak/hole/e Å^−3^	0.95/−1.10	1.09/−1.05

**Table 4:** The selected bond distances (Å) and angles (deg) for compounds **1-2**.

Compound **1**					
Pb1-I1	3.3015 (5)	Pb1-I2	2.9931 (5)	Pb1-I3	3.2378 (5)
Pb1-I4	3.1758 (5)	Pb1-I6^1^	3.3566 (5)	Pb2-I1^2^	3.3261 (5)
Pb2-I3^3^	3.2566 (5)	Pb2-I4	3.2066 (5)	Pb2-I5	2.9998 (5)
Pb2-I6	3.3275 (5)	I1-Pb1-I6^1^	94.147 (13)	I2-Pb1-I1	90.717 (13)
I2-Pb1-I3	91.864 (15)	I2-Pb1-I4	94.913 (14)	I2-Pb1-I6^1^	88.327 (14)
I3-Pb1-I1	85.429 (13)	I3-Pb1-I6^1^	179.537 (15)	I4-Pb1-I1	174.300 (14)
I4-Pb1-I3	95.263 (15)	I4-Pb1-I6^1^	85.140 (14)	I1^2^-Pb2-I6	91.846 (13)
I3^3^-Pb2-I1^2^	178.799 (15)	I3^3^-Pb2-I^6^	88.579 (14)	I4-Pb2-I1^2^	82.350 (13)
I4-Pb2-I3^3^	97.253 (14)	I4-Pb2-I6	174.008 (16)	I5-I2-I1^2^	91.964 (14)
I5-Pb2-I3^3^	89.166 (14)	I5-Pb2-I4	89.512 (14)	I5-I2-I6	89.201 (14)
Pb1-I1-Pb2^2^	137.703 (16)	Pb1-I3-Pb2^4^	128.609 (17)	Pb1-I4-Pb2	87.013 (12)
Pb2-I6-Pb1^1^	135.438 (16)				
Compound **2**					
Pb1-I1	3.1687 (6)	Pb1-I1^1^	3.1688 (6)	Pb1-I3^1^	3.3441 (6)
Pb1-I3	3.3441 (6)	Pb1-I2^1^	3.2365 (6)	Pb1-I2	3.2364 (6)
Pb2-I1^1^	3.3841 (7)	Pb2-I3	3.2696 (7)	Pb2-I2^2^	3.3502 (7)
Pb2-I4	2.9499 (7)	Pb2-I5	3.0913 (6)	I1-Pb1-I1^1^	180.0
I1-Pb1-I3^1^	84.097 (15)	I1^1^-Pb1-I3^1^	95.901 (16)	I1-Pb1-I3	95.903 (16)
I1^1^-Pb1-I3	84.098 (15)	I1-Pb1-I2	92.692 (19)	I1-Pb1-I2^1^	87.309 (19)
I1^1^-Pb1-I2^1^	92.690 (19)	I1^1^-Pb1-I2	87.309 (19)	I3-Pb1-I3^1^	180.0
I2^1^-Pb1-I3^1^	79.946 (16)	I2^1^-Pb1-I3	100.054 (16)	I2-Pb1-I3^1^	100.054 (16)
I2-Pb1-I3	79.946 (16)	I2-Pb1-I2^1^	180.0	I3-Pb2-I1^1^	81.954 (17)
I3-Pb2-I2^2^	163.585 (14)	I2^2^-Pb2-I1^1^	81.683 (18)	I4-Pb2-I1^1^	88.270 (17)
I4-Pb2-I3	88.450 (15)	I4-Pb2-I2^2^	89.767 (16)	I4-Pb2-I5	85.572 (19)
I5-Pb2-I1^1^	173.831 (14)	I5-Pb2-I3	98.223 (19)	I5-Pb2-I2^2^	97.91 (2)
Pb1-I1-I2^1^	88.903 (19)	Pb2-I3-Pb1	87.927 (15)	Pb1-I2-Pb2^2^	109.55 (2)

## Data Availability

All data generated or analyzed during this study are included in this article.

## Sample Availability

Samples of the compounds are not available from the authors.
